# Inducible clindamycin and methicillin resistant *Staphylococcus aureus* in a tertiary care hospital, Kathmandu, Nepal

**DOI:** 10.1186/s12879-017-2584-5

**Published:** 2017-07-11

**Authors:** R. P. Adhikari, S. Shrestha, A. Barakoti, R. Amatya

**Affiliations:** Department of Microbiology, Nepal Medical College and Teaching Hospital, Jorpati, Kathmandu Nepal

**Keywords:** *Staphylococcus aureus*, MRSA, Inducible clindamycin resistance, Nepal

## Abstract

**Background:**

*Staphylococcus aureus,* an important nosocomial pathogen, is frequently associated with infections in human. The management of the infections by it especially methicillin resistant ones is often difficult because methicillin resistant *S. aureus* is usually resistant to multiple antibiotics. Macrolide-lincosamide streptogramin B family of antibiotics is commonly used to treat such infections as an alternative to vancomycin.

**Methods:**

This study was conducted over the period of one and half year from November 2013–April 2015 in Microbiology laboratory of Nepal Medical College and Teaching Hospital, Kathmandu, Nepal to find the incidence of different phenotypes of MLS_B_ resistance among *S. aureus* from clinical samples and their association with methicillin resistance. Two hundred seventy isolates of *S. aureus* were included in the study. Methicillin resistance was detected by cefoxitin disc diffusion method and inducible clindamycin resistance by erythromycin and clindamycin disc approximation test (D-test).

**Results:**

Of the 270 clinical isolates of *S. aureus,* 25.1% (68/270) were MRSA. Erythromycin and clindamycin resistance was seen in 54.4% (147/270) and 41.8% (113/270) isolates respectively. Resistance to erythromycin and clindamycin were higher in MRSA as compared to MSSA (erythromycin-resistance: 88.2% Vs 39.1% and clindamycin-resistance: 79.4% Vs 41.8%). The overall prevalence of _i_MLS_B_ and _c_MLS_B_ phenotype was 11.48% (31/270) and 29.25% (79/270) respectively. Both _i_MLS_B_ and _c_MLS_B_ phenotypes predominated in MRSA strains.

**Conclusions:**

Detection rate of MRSA in our study shows the necessity to improve in healthcare practices and to formulate new policy for the control of MRSA infections. Clindamycin resistance in the form of _i_MLS_B_ and _c_MLS_B_ especially among MRSA emphasizes the need of D-test to be performed routinely in our set up while using clindamycin as an alternative choice to anti-staphylococcal antibiotics like vancomycin and linezolid in the treatment of staphylococcal infections.

## Background

*Staphylococcus aureus*, one of the most common nosocomial and community-acquired pathogens has now emerged as an ever-increasing problem due to its increasing resistance to several antibiotics. In *Staphylococcus* spp., penicillin and methicillin resistance was first recognized in 1944 and 1961 A.D. respectively [1]. Emerging resistance to methicillin in this organism has left us with very few therapeutic alternatives to treat the infections caused by them. Clindamycin in macrolide-lincosamide streptogramin B (MLS_B_) family of antibiotics serves as one such alternative for treating both methicillin susceptible *S. aureus* (MSSA) and methicillin resistant *S. aureus* (MRSA) infections, due to its excellent pharmacokinetic properties [2]. However, widespread use of this antibiotic has led to a large number of staphylococcal strains resistant to it [3]. Resistance to MLS_B_ antibiotics occur by many different mechanisms. The most common mechanism for such resistance is target site modification mediated by *erm* genes, which can be expressed either constitutively (_c_MLS_B_ phenotype) or inducibly (_i_MLS_B_ phenotype). The *erm* genes codes for methylase enzyme which methylates and alters the target site of MLS_B_ antibiotics i.e. the 23S ribosomal RNA [4].

It is very difficult to detect the inducible clindamycin resistance in the routine laboratory as they appear erythromycin-resistant and clindamycin sensitive in vitro when not placed adjacent to each other. In such cases, in vivo therapy with clindamycin may select constitutive *erm* mutants leading to clinical therapeutic failure. In case of another mechanism of resistance mediated through *msr*A genes i.e. efflux of antibiotic, staphylococcal isolates appear erythromycin-resistant and clindamycin-sensitive both in vivo and in vitro and the strain do not typically become clindamycin resistant during therapy [5]. Thus to avoid clinical therapeutic failure in the resistance case mediated by *erm* gene, it is very important to detect inducible clindamycin resistance phenotypes in vitro which can be made by erythromycin-clindamycin disc approximation test (D-test) as its sensitivity was found 100% in different studies when compared with *erm* and *msr* gene detection by polymerase chain reaction [6–8]. There is a wide variation in the rate of inducible clindamycin resistance in different places [9–13]. In Nepal, very few reports on prevalence of inducible clindamycin resistance among *S. aureus* have been published [14, 15]. This study was conducted to determine the prevalence of inducible clindamycin resistance among clinical *S. aureus* isolates and also to study their association with MRSA in our set up.

## Methods

A descriptive cross sectional study was conducted over the period of one and half year (November 2013–April 2015) in the microbiology laboratory of Nepal Medical College and Teaching Hospital (NMCTH), Kathmandu, Nepal. The study was done in 270 non-repeated isolates of *S. aureus* from clinical specimens (pus, blood, urine, sputum and body fluids) from both gender and all age groups of patients attending NMCTH.

### Isolation and identification

All specimens were inoculated on sheep blood agar, MacConkey agar without crystal voilet (Hi-Media-India) and incubated at 37 °C aerobically for 24 h. Identification of *S. aureus* was first done using colony morphology on 5% sheep blood agar. Cream to golden yellow colonies with or without haemolysis were further identified using Gram stain, catalase test and coagulase test by standard microbiological techniques [16].

### Antibiotic susceptibility test

Antibiotic susceptibility were studied by modified Kirby Bauer’s disc diffusion method on Mueller Hinton Agar plates (12 cm diameter) using ampicillin (10 μg), cotrimoxazole (1.25/23.75 μg), ciprofloxacin (5 μg), vancomycin (30 μg), cephalexin (30 μg) and gentamycin (10 μg) discs. Cefoxitin (30 μg) for the detection of methicillin resistance and erythromycin (15 μg), clindamycin (2 μg) discs (Hi-media-India) at 15 mm apart were also used on same plate for the detection of inducible clindamycin resistance as per CLSI guidelines [17].

### Detection of methicillin resistance

Isolates with cefoxitin zone size ≥22 mm were considered methicillin susceptible and those with ≤21 mm were considered methicillin resistant.

### Detection of clindamycin resistance

Clindamycin resistance was detected as:

1. Inducible resistance phenotypes (_i_MLS_B_): Resistant to erythromycin and having a clindamycin zone ≥21 mm with a D-shaped zone.
2. Constitutive resistance phenotypes (_c_MLS_B_): resistant to both erythromycin and clindamycin
3. MS phenotype: Isolates resistant to erythromycin and susceptible to clindamycin without D-zone [17].

*S. aureus* ATCC 25923 was used to perform quality control. Separate in house selected *S. aureus* strains that demonstrated the above clindamycin resistance phenotypes were also used in quality control.

### Data analysis

Data was analyzed using SPSS 17.0. Chi-square test was used for analyzing categorical variables (*P* < 0.05 was considered significant).

## Results

From both the in-patients and out-patients, a total of 16,789 specimens (urine 7970, blood 4905, sputum 1591, pus 1504 and body fluids 819) were processed. Of 270 isolates of *Staphylococcus aureus*, 150 were from male patients and 120 from female patients. The isolates obtained were 147 (54.4%), 60 (22.2%), 38 (14.0%), 20 (7.4%) and 5 (1.85%) from pus, blood, sputum, urine and body fluids respectively. The highest positivity rate among the processed samples was found in pus sample (9.8%) followed by sputum (2.4%), blood (1.2%), body fluids (0.6%) and urine (0.2%). The age distribution of the isolates is shown in Table 1.

**Table 1:** Distribution of clinical isolates of *Staphylococcus aureus* according to the age of patients (*n* = 270)

Age of patients in years	Number of isolates (%)
0–10	44 (16.3)
11–20	41 (15.2)
21–30	65 (24.1)
31–40	50 (18.5)
41–50	24 (8.9)
51–60	21 (7.8)
61–70	16 (5.9)
71–80	9 (3.3)
Total	270 (100)

Among the antibiotics tested all the isolates were susceptible only to vancomycin. Gentamycin was still found to have better action as compared with other antibiotics. However, most of the isolates were resistant to commonly used antibiotics Table 2.

**Table 2:** Antibiogram of *Staphylococcus aureus* (*n* = 270)

Antimicrobial agents	Resistant isolates (%)
Ampicillin	214 (79.2)
Erythromycin	147 (54.4)
Cotrimoxazole	146 (54.0)
Clindamycin	113 (41.8)
Ciprofloxacin	74 (27.4)
Cephalexin	70 (25.9)
Cefoxitin	68 (25.1)
Gentamycin	21 (7.8)
Vancomycin	00

**Fig. 1:**
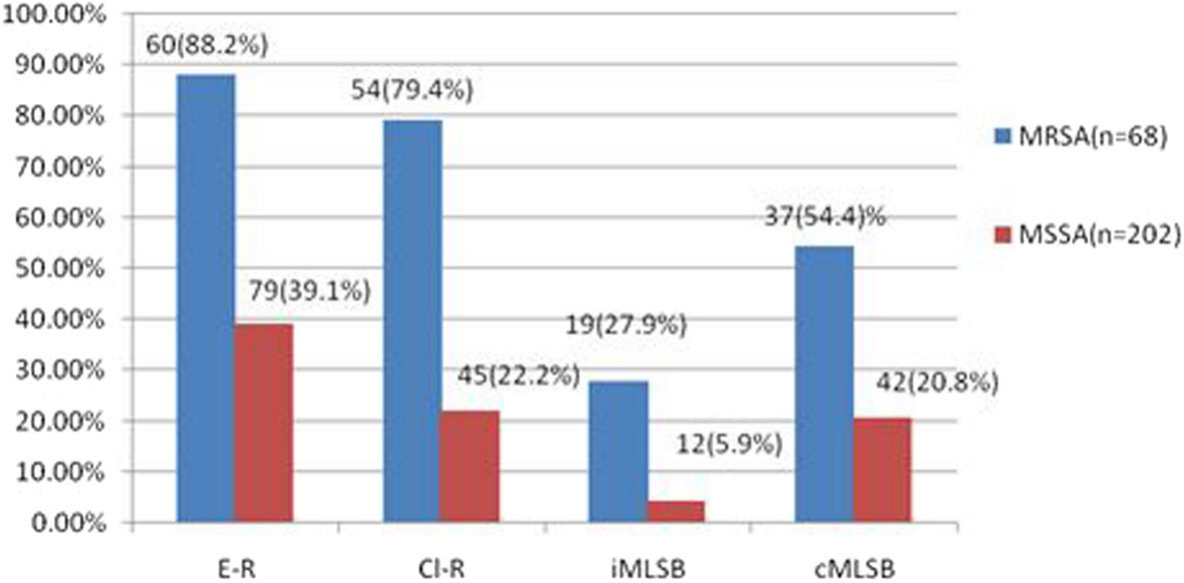
Comparision of erythromycin, clindamycin, _i_MLS_B_ and _c_MLS_B_ resistance among MRSA and MSSA

Of the 270 clinical isolates of *S. aureus* 25.1% (68/270) were MRSA. Erythromycin and clindamycin resistance was seen in 54.4% (147/270) and 41.8% (113/270) isolates respectively. Resistance to erythromycin and clindamycin were higher in MRSA as compared to MSSA (E-R: 88.2% Vs 39.1% and Clin-R: 79.4% Vs 22.2%) (*p* value = 0.006) (Fig. 1). Erythromycin sensitive and clindamycin resistance was detected in 3 MRSA isolates. The overall prevalence of _i_MLS_B_, _c_MLS_B_ and MS phenotypes was 11.48% (31/270), 29.25% (79/270) and 13.7% (37/270) respectively. Both _i_MLS_B_ and _c_MLS_B_ phenotypes predominated in MRSA strains (*p* value = 0.002) (Fig. 1 and Table 3). Among 147 erythromycin resistant isolates, 12.9% iMLSB, 25.2% _c_MLS_B_ and 4.76% MS phenotype were MRSA (Table 4).

**Table 3:** Clindamycin susceptibility patterns among MRSA and MSSA

		MRSA (%)	MSSA (%)	Total (%)
		*N* = 68	*N* = 202	*N* = 270
E-S	E- S, CI-S	2 (2.9)	118(58.4%)	120 (44.5)
(*n* = 123)	E- S, CI-R	3(4.4)	x	03(1.1)
E-R (*n* = 147)	E- R, CI-S (_i_MLS_B_)	19(27.9)	12 (5.9)	31(11.5)
	E-R,CI-R (_c_MLS_B_)	37 (54.4)	42 (20.8)	79 (29.2)
	E-R, CI-S (MS Phenotype)	7 (10.3)	30 (14.8)	37(13.7)

*S* sensitive

**Table 4:** Clindamycin susceptibility pattern among erythromycin resistant isolates (*n* = 147)

	MRSA	MSSA	Total
_i_MLS_B_	19 (12.9%)	12 (8.1%)	31 (21.0%)
_c_MLS_B_	37 (25.2%)	42(28.5%)	79 (53.4%)
MS Phenotype	7 (4.76%)	30 (20.4%)	37 (25.17%)

## Discussion

The proportion of MRSA has increased worldwide since last two decades. Its prevalence varies markedly across different countries and among hospitals of the same country [18, 19]. Improper infection prevention practices in the hospital set up, indiscriminate use of antibiotics, intravascular catheterization, hospitalization in intensive care unit etc. contribute in the emergence of MRSA [20]. This study showed prevalence rate of 25.1% which is similar to the study done in eastern part of Nepal [21] India [2] and other part of the world [9]. However higher rates of MRSA were also noted in other studies conducted in Nepal [14, 22–24] and other countries [10, 11, 13, 18]. These variations could be due to the differences in the circulating clones or due to the variations in infection prevention practices and trends of antibiotics prescription in different hospital set up.

In this study the prevalence of _i_MLS_B_ among *S. aureus* was found to be 11.48% which is similar to that reported by Ansari et al. (12.4%) [24], Sah et al. (12.1%) [14] from Nepal and Govindan et al. (11.6%) from India [12]. Varying prevalence rates of _i_MLS_B_ have been reported in different other studies; 18.2% from Nepal [25] 28.6% from Iran [11], 20.7% [13] and 24.3% [10] from India. Higher _i_MLS_B_ prevalence of 37.5% from India [26] and 91% from Japan [27] has also been reported. A comparatively low prevalence of inducible resistance in this study could be due to the geographical variations of circulatory clones.

In this study, erythromycin resistance (88.2% Vs 54.4%) and clindamycin resistance (79.4% Vs 41.8%) both was significantly higher in MRSA than among MSSA (*p* value = 0.006). Similarly both the constitutive and inducible clindamycin resistance phenotypes were significantly higher in MRSA (54.4% and 27.9%) than MSSA (20.79% and 4.95%) respectively (*P* = 0.002) which is similar to other reports [2, 12, 25]. Molecular studies have shown that some SCCmec elements on MRSA carry transposon Tn554 which contains the gene *erm*A mediating MLS resistance resulting higher rate of resistance to MLS antimicrobial agents [4]. However, higher incidence of _i_MLS_B_ among MSSA was reported by Schreckenberger et al. [28] and Levin et al. [29].

Even though the overall prevalence of inducible clindamycin resistance among the isolates was found to be low in our set up, this study showed higher percentage of resistance to erythromycin and clindamycin among MRSA as compared to other studies [9, 13, 14]. This indicates that there is wide use of erythromycin and clindamycin for the treatment of staphylococcal infections in our set up, as wide consumption of macrolides results emergence of macrolide resistant *Staphylococcus* species due to selective pressure [1]. As this resistant patterns can be decreased by reduction in the use of macrolides [1] this study emphasizes the need to do likewise in our set up to reserve it as an alternative drug of choice for treating infection caused by MRSA. This study showed only 4.76% of MRSA among the erythromycin resistant isolates as MS phenotype (E-R, Clin-S) which means clindamycin can be used as treatment option only for less number of MRSA which are erythromycin resistant. So there is a least chance of clinical efficacy of clindamycin while treating erythromycin resistant MRSA infections as an alternative to vancomycin. These findings further emphasize the need of D- test to be performed routinely in our set up to avoid clinical failure while using clindamycin as an alternative to anti-MRSA antibiotics like vancomycin and linezolid.

## Conclusions

Staphylococcus, particularly MRSA, has emerged as a major global health problem both in community and hospitals. Since these are resistant to the commonly used antibiotics, there is a need for the development, adoption, and enforcement of appropriate control policies in our hospital settings. Regular surveillance of hospital-associated infections including monitoring of antimicrobial (especially vancomycin) susceptibility pattern of MRSA and formulation of a definite antimicrobial policy may be helpful in reducing the incidence of these infections. A further study of MRSA may be conducted for the epidemiological mapping of these infections. Clindamycin resistance in the form of _i_MLS_B_ and _c_MLS_B_ limits the therapeutic options for MRSA to the antibiotics like linezolid and vancomycin. Therefore to identify these resistance mechanisms phenotypically, D-test should be routinely performed that will help us in guiding the clinicians regarding the judicious use of clindamycin.

## Abbreviations

Cl: Clindamycin
_c_MLS_B_: Constitutive clindamycin resistance phenotypes
E: Erythromycin
_i_MLS_B_: Inducible clindamycin resistance phenotypes
MLS_B_: Macrolide-lincosamide streptogramin B
MRSA: Methicillin resistant *Staphylococcus aureus*
MSSA: Methicillin sensitive *Staphylococcus aureus*
NMCTH: Nepal Medical College and Teaching Hospital
R: Resistant
RNA: Ribonucleic acid
μg: Microgram
